# Images in infectious diseases: milker’s nodule with erythema multiforme after calf bite in a 23-year-old patient

**DOI:** 10.1007/s15010-025-02475-2

**Published:** 2025-02-12

**Authors:** Benjamin T. Schleenvoigt, Christine Kletta, Christine Zollmann, Stefan Hagel, Stefan Glöckner, Eva Krause, Janine Michel, Carlotta Helbig, Andrea Vanegas-Ramirez

**Affiliations:** 1 Institute of Infectious Diseases and Infection Control, Jena University Hospital/Friedrich-Schiller-University, Jena, Germany; 2 General Practitioner, Jena, Germany; 3 Praxis für Venen- und Hauterkrankungen, Jena, Germany; 4 Institute of Medical Microbiology, Jena University Hospital/Friedrich-Schiller-University, Jena, Germany; 5 Centre for Biological Threats and Special Pathogens, Unit Highly Pathogenic Viruses (ZBS 1), Robert Koch Institute, Berlin, Germany; 6 Jena University Hospital/Friedrich-Schiller-University, Jena, Germany; 7 Department of Dermatology, Venerology and Allergology, Bundeswehrkrankenhaus Hamburg, Hamburg, Germany; 8 Tropical Dermatology Outpatient Care Center for Tropical Medicine, Bernhard Nocht Institute for Tropical Medicine, University Medical Center Hamburg-Eppendorf, 20359 Hamburg, Germany

A 23-year-old patient presented to the general practitioner´s (GP’s) office with a hard nodule on her right index finger. This had been preceded by a calf bite in the same place 4 weeks earlier. The wound had initially healed within a few days without sequelae. Clinically, the nodule was approximately 1 cm in size, with a whitish margin and central hemorrhagic erythema (Fig. 1).

**Fig. 1:**
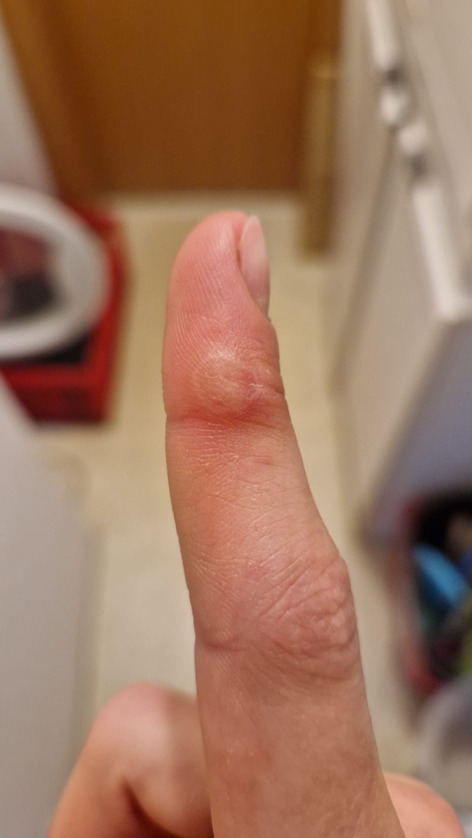
Nodule on the right index finger, 4 weeks after the calf bite

The puncture remained dry, and the swab showed no bacterial growth. After starting empirical antibiotic therapy with amoxicillin/clavulanate, a macular pruritic rash occurred, beginning on both distal extremities and spreading to the trunk (Figs. 2 and 3). The patient also reported a general malaise, pain in her limbs and loss of appetite.

**Fig. 2:**
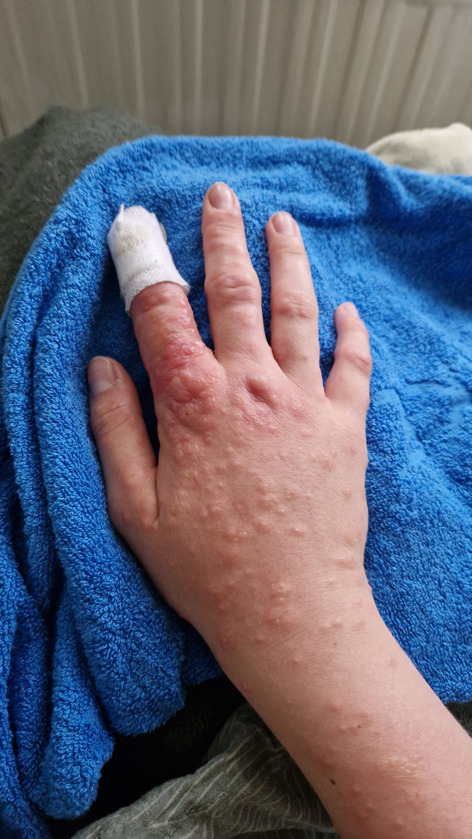
Macular pruritic rash after starting antibiotic treatment

**Fig. 3:**
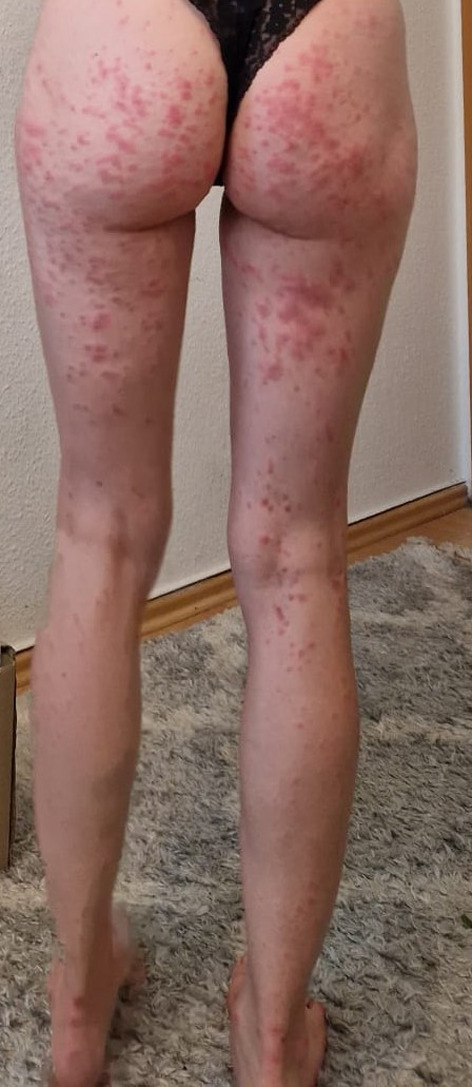
Macular pruritic rash after starting antibiotic treatment

Amoxicillin/clavulanate was discontinued after 6 days, on the assumption that the rash was drug-related. In view of the previous animal contact, a swab for poxvirus analysis was sent to the Robert Koch Institute, and the PCR test for parapoxviruses returned a positive result [1]. The sample was identified as pseudocowpox virus by Sanger sequencing of the *B2L* gene. Furthermore, parapoxvirus specific IgM (1:1280) and IgG (1:320) were detected by immunoflourescence assay in a serum sample taken 7 weeks after the bite. In the context of the diagnosis of pseudocowpox, the rash was most likely to be classified as infection-induced erythema multiforme [2].

Topical steroids (mometasone furoate cream 1 mg/g, applied twice daily for two weeks) and systemic steroids (methylprednisolone: 40 mg on day 1, 20 mg for 2 days, 10 mg for 2 days, and 5 mg for 2 days) were administered, complemented by topical antibiotic treatment (Fusidic acid cream) as a preventive measure [3]. Analgesic treatment (Ibuprofen 600 mg) was used as needed. Within the next two weeks, the exanthema gradually subsided, and the nodule on the index finger healed without scarring.

## Data Availability

No datasets were generated or analysed during the current study.
